# A Multigene Signature for Prognostic Stratification of Nasopharyngeal Carcinoma

**DOI:** 10.3390/cancers18081197

**Published:** 2026-04-09

**Authors:** Yingying Liang, Zhiwen Mo, Muy-Teck Teh

**Affiliations:** 1 Department of Radiation Oncology, Guangzhou Institute of Cancer Research, The Affiliated Cancer Hospital, Guangzhou Medical University, Guangzhou 510095, China; yingyingliang@gzhmu.edu.cn (Y.L.); mozhiwen308@163.com (Z.M.); 2 Centre for Oral Immunobiology and Regenerative Medicine, Institute of Dentistry, Barts and the London School of Medicine and Dentistry, Queen Mary University of London, London E1 2AT, UK

**Keywords:** molecular diagnostics, nasopharyngeal carcinoma, qMIDS, squamous cell carcinoma, mRNA biomarkers, Epstein–Barr virus (EBV)-independent detection, risk stratification

## Abstract

**Background**: We previously pioneered a multigene mRNA test, qMIDS^V2^, validated through an international multicohort study with geographically and ethnically diverse oral squamous cell carcinoma (OSCC) patients from Europe and Asia. This study aimed to repurpose the qMIDS^V2^ test for nasopharyngeal carcinoma (NPC). A molecular test independent of Epstein–Barr virus (EBV) status would be clinically useful for risk stratification in NPC patients with undetectable or low levels of EBV. **Methods**: This study investigated a Chinese cohort of 62 participants (18 donated normal nasopharyngeal mucosa (NPM) and 44 donated NPC tissue samples). Messenger RNA levels of 16 genes in each sample were quantified using the qPCR method, and an algorithm computed a malignancy index for cancer risk stratification. **Results**: We identified a unique 10-gene panel (containing eight target genes, namely NEK2, INHBA, FOXM1, TOP2A, BIRC5, CXCL8, NR3C1, and IVL, relative to two reference genes, YAP1 and POLR2A, collectively named qMIDS^NPC^) that demonstrated the best overall diagnostic performance in segregating NPM from NPC, with AUC = 0.909 and positive/negative predictive values of 91% PPV and 78% NPV, respectively. Furthermore, we demonstrated prognostic value of qMIDS^NPC^ in segregating NPM from NPC stage III + IV, with AUC = 0.936, 92% PPV, and 84% NPV. **Conclusions**: Here, we present a simple qPCR-based 10-gene mRNA test, qMIDS^NPC^, with potential clinical utilities for rapid (1 h) prognostic stratification of NPC. Further studies involving geographically and ethnically independent NPC cohorts would be needed to validate the clinical use of qMIDS^NPC^ in non-endemic NPC populations.

## Simple Summary

Nasopharyngeal carcinoma (NPC) remains difficult to diagnose early, particularly in patients with low or undetectable plasma Epstein–Barr virus (EBV) DNA. In this study, we repurposed a previously validated 16-gene head and neck cancer mRNA assay (qMIDS^V2^) to evaluate its utility in NPC. Using nasopharyngeal tissue samples from 62 individuals, we quantified mRNA expression of 16 biomarkers and systematically optimized gene-panel combinations. A unique 10-gene signature, termed qMIDS^NPC^, demonstrated the highest diagnostic performance, distinguishing normal nasopharyngeal mucosa from NPC with AUC = 0.909, 91% PPV, and 78% NPV. qMIDS^NPC^ also exhibited strong prognostic capability, segregating advanced NPC stages (III–IV) with AUC = 0.936, 92% PPV, and 84% NPV. Importantly, the assay accurately detected both EBV-negative and EBV-positive NPC, confirming its EBV-independent biomarker value. This rapid, 1 h qPCR-based test shows promise for risk stratification and clinical management of NPC, warranting validation in larger multi-ethnic cohorts.

## 1. Introduction

Nasopharyngeal carcinoma (NPC) is a type of squamous cell carcinoma that arises from mucosal epithelium of the nasopharynx, predominantly found within the pharyngeal recess (fossa of Rosenmüller). Global geographical distribution of NPC is unique, with endemic cases in Southern China, Southeast Asia, Northern Africa, Greenland, and Alaska, which are invariably associated with previous Epstein–Barr virus (EBV) infection [1]. A study based on the Global Burden of Disease Study 2019 found a 45% increase in the global incidence of NPC between 2009 and 2019 (from 121.65  ×  10^3^ cases in 2009 to 176.50  ×  10^3^ cases in 2019); meanwhile, the mortality and disability-adjusted life years (DALYs) have been decreasing [2].

Plasma EBV DNA is a well-established biomarker for NPC detection, risk stratification, and disease surveillance [3,4]. However, 15–40% of NPC patients show negative EBV DNA levels, especially in regions where NPC is less prevalence [3,5], and one study estimated that using plasma EBV DNA alone as a population-screening tool in endemic population could potentially miss 60.0%, 23.0%, 14.5%, and 5.0% of NPC stage I, II, III, and Iva, risking delayed diagnosis and missed treatment opportunities [5]. Furthermore, a study using plasma EBV levels for disease surveillance to predict NPC recurrence has shown that positive predictive values in patients with low EBV levels (<500 copies/mL) were poor (PPV of 2.6–34.3%, with test sensitivity of 16.9–23.1%) [4]. Emerging evidence has shown that SARS-CoV-2 infection could result in unreliable fluctuations in EBV levels, hence rendering EBV DNA alone insufficient for monitoring NPC relapse after radical therapy [6]. Hence, there is a unmet clinical need for alternative EBV-independent biomarkers that could be used for both NPC diagnosis and prognosis in patients with low or undetectable EBV levels [3,5].

Recent advances in multimodal integrated technologies involving single-cell multiomics and spatial precision imaging have produced high-resolution data capable of untangling heterogeneity and highly complex biomolecular networks from a myriad of clinical sample types; however, clinical translation of these high-dimensional biological data into practical cost-effective clinical tools remains a challenge for widespread use, especially in resource-poor settings [7,8,9,10,11]. At present, histopathology remains the current standard for tumor classification, but it relies on a pathologist’s subjective interpretation of microscopic structures of cells and tissue morphology as the basis for tumor diagnosis and grading. The major limitations of histopathology are that it is time-consuming (usually several days for tissue fixing and reporting) and the result is subject to pathologists’ interpretation [12]. In addition, the presence of dysplasia is often missed because molecular changes indicative of malignant transformation do not necessarily produce clinically or histopathologically detectable changes [13]. Because this diagnostic approach is often not reliable for detecting early malignancy [13,14], there is a need for a practical, reproducible, objective, and quantitative molecular method for the detection and management of NPC patients.

We previously pioneered a multi-gene qPCR test, the quantitative Malignancy Index Diagnostic System (qMIDS), requiring only 1 mm^3^ of minimally invasive biopsy tissues for diagnosis. The first version of qMIDS^V1^ was initially validated on UK and Norwegian head and neck SCC (HNSCC) samples [15] and subsequently on Chinese oral SCC (OSCC) samples [16]. The recently updated version (qMIDS^V2^) with an improved gene panel has been validated through an international multicohort study involving 535 tissue specimens from geographically and ethnically diverse oral cancer patients from the UK, India, and China [17]. The qMIDS test involves measuring the mRNA expression levels of 16 genes (14 target + two reference; tested in both fresh frozen and archival FFPE samples) from each sample, and an algorithm calculates a malignancy index for cancer detection and risk stratification [15,17]. In this study, we investigated the molecular profiles based on the 16 genes and their clinical potential for NPC detection and risk stratification in a Chinese NPC cohort.

## 2. Materials and Methods

### 2.1. Clinical Samples

**Figure 1:**
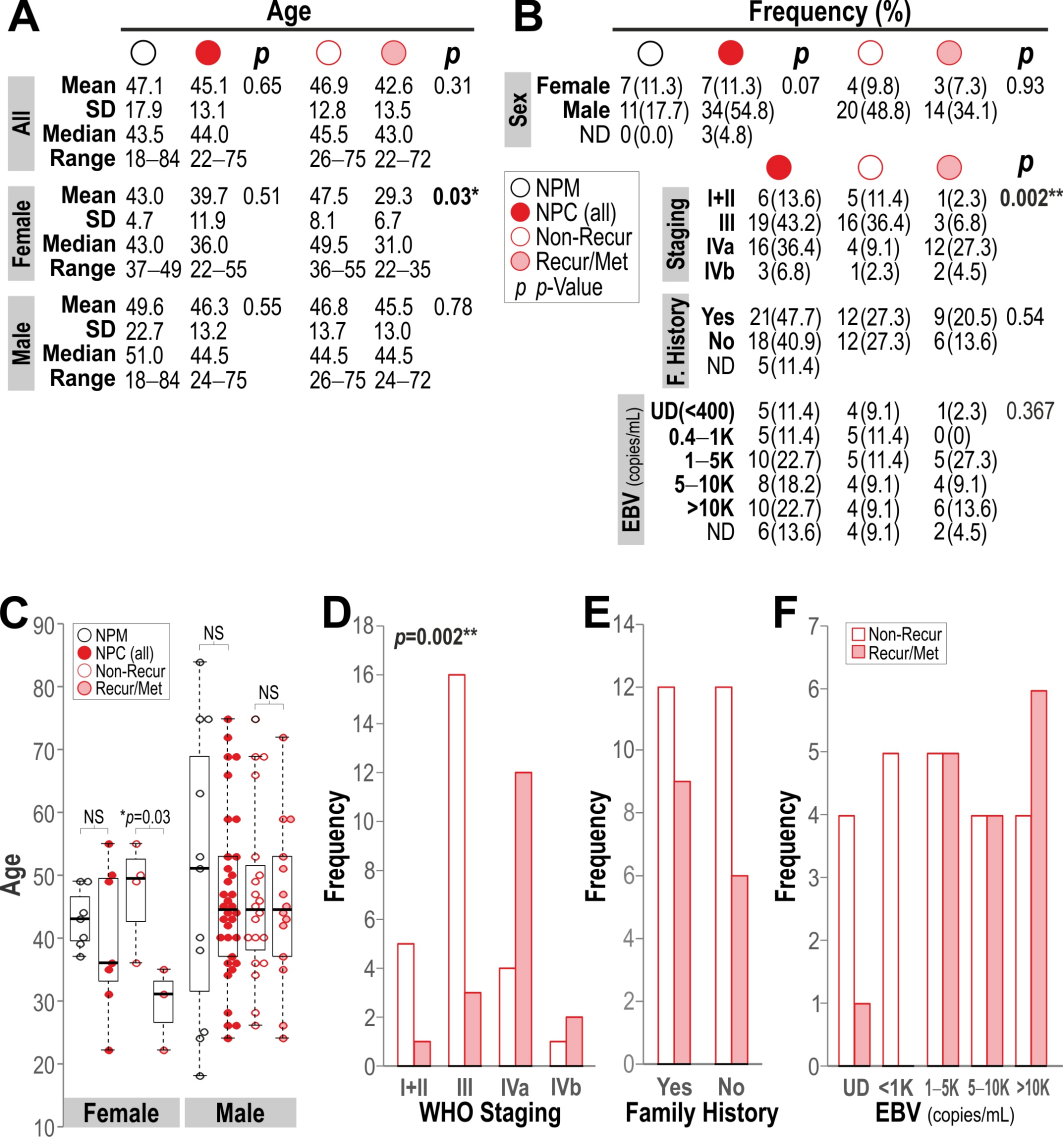
Participants demographics and clinicopathological characteristics of NPC. (**A**) Age distributions in healthy participants donating normal nasopharyngeal mucosa (NPM) and nasopharyngeal carcinoma (NPC), with subdivisions by sex (female or male; data plotted in panel (**C**)) and tumor recurrence status (non-recurrence or recurrence/metastatic tumors). Student’s *t*-test was applied between two groups of data, with corresponding *p*-value as indicated. * *p* < 0.05 indicates statistically significant difference in age of female NPC patients with non-recurring and recurring/metastatic tumor. NS, not significant. (**B**–**F**) Frequency of each sample type as indicated according to sex, tumor staging (data plotted in panel (**D**)), family history (data plotted in panel (**E**)), and EBV DNA levels (data plotted in panel (**F**)). UD, EBV undetectable or less than 400 copies/mL. ND, not determined or missing. Chi-square test was applied between two groups, with corresponding *p*-values indicated within the panel (**B**). ** *p* < 0.01 indicates statistically significant difference between tumor staging of non-recurring and recurring/metastatic tumors.

The use of human tissue was approved by the relevant Research Ethics Committees at each institution: Queen Mary University of London (QMERC20.142), Affiliated Cancer Hospital and Institute of Guangzhou Medical University, Guangzhou, China (GMU No.32/2019). All tissue samples were collected according to local ethical committee-approved protocols, and informed patient consent was obtained from all participants. In this retrospective diagnostic case–control study cohort of 62 ethnically Chinese individuals, 18 healthy participants donated normal nasopharyngeal mucosa (NPM) and 44 donated nasopharyngeal carcinomas (NPC). Nasopharyngeal tissue biopsy samples were collected during nasoendoscopy procedure. To accomplish this, a medical professional inserted a small, flexible tube equipped with a camera into the nasopharynx. Upon identifying the tumor, a specialized tool was passed through the endoscope to extract a tissue sample. The majority of the collected tissue was then sent for pathological examination under a microscope. Clinico-histopathological reports of all tissue samples were obtained from collaborating clinicians at the Affiliated Cancer Hospital of Guangzhou Medical University. NPC tumor-stage diagnosis was based on the World Health Organization (WHO) classification criteria [18]: keratinizing squamous cell carcinoma (type 1); and non-keratinizing carcinoma, which is further subdivided into differentiated (type 2) and undifferentiated (type 3) subtypes. The study included a total follow-up duration of five years. The clinicopathological characteristics of the cohort are summarized in Figure 1.

Inclusion and exclusion criteria: Only primary disease adult patients (≥18 years of age) with clinicopathologically confirmed NPM and NPC were included, and patients were excluded if they met the following exclusion criteria: (1) patients diagnosed with nasopharyngeal sarcoma or tuberculosis; and (2) samples that failed to provide sufficient quality RNA to enable SYBR green fluorescent detection of at least 6 target genes in the initial qMIDS^V2^ 16-gene assay [17], and the 2 reference genes by RT-qPCR quantification.

Each fresh tissue sample (1–5 mm^3^) was immediately preserved in RNA*Later* and later subjected to TRIzol-based total RNA extraction (Monach Total RNA Miniprep Kit, New England Biolabs Ltd., Hertfordshire, UK). One microgram of total RNA was converted to cDNA (One Step PrimeScript™ RT-PCR Kit, Takara Bio Inc., England, UK) prior to storage at −20 °C until use. All samples were pseudo-anonymized and tested blindly to ensure that the qMIDS assays were performed objectively. All samples provided RNA of adequate quality and quantity for the qMIDS^V2^ 16-gene assay; thus, no samples were excluded from analysis.

EBV DNA detection involves measuring the presence of Epstein–Barr virus DNA in patient blood sample by quantitative PCR with detection of EBNA1-a segment of EBV genome, and the limit of detection was 400 copies per mL plasma in this study.

### 2.2. The qMIDS Assay

The quantitative Malignancy Index Diagnostic System (qMIDS) assay methodology was performed as described previously [15,17,19,20,21], with minor modifications. Briefly, the present assay format involves using qPCRBIO SyGreen Blue Mix Lo-ROX (PCR Biosystems Ltd., England, UK, PB20.15-51) for two-step relative quantification of 14 target genes (*HOXA7, CENPA, NEK2, DNMT1, INHBA, FOXM1, TOP2A, BIRC5, MMP13, CXCL8, IVL, NR3C1, CBX7*, and *S100A16*) and 2 reference genes (*YAP1* and *POLR2A*), performed using a 384-well format LightCycler 480 qPCR system (Roche Diagnostics Ltd., West Sussex, UK) based on our previously published protocols [15,17,19,20,21], which are MIQE-compliant [22]. Thermocycling begins with 45 °C for 10 min (for reverse transcription), followed by 95 °C for 30 s prior to 45 cycles of amplification at 95 °C for 1 s, 60 °C for 1 s, 72 °C for 1 s, and 78 °C for 1 s (data acquisition). A ‘touch-down’ intervention (66 °C starting temperature with a stepwise reduction of 0.6 °C/cycle; 8 cycles) was introduced prior to the amplification step to maximize primer specificity and reduce primer dimerization. Melting analysis (95 °C for 30 s, 75 °C for 30 s, and 75–99 °C at a ramp rate of 0.57 °C/s) was performed at the end of qPCR amplification to validate single-product amplification in each well [17]. Relative quantification of mRNA transcripts was calculated based on an objective method using the second derivative maximum algorithm [23] (Roche). All qPCR primer sequences, specificity, and metadata of all the 16 genes were published previously [17]. Each target gene was normalized to two stable reference genes (*YAP1* and *POLR2A*), validated previously [19] to be amongst the most stable reference genes across a wide variety of primary human epithelial cells, dysplastic and squamous carcinoma cell lines, using the geNORM algorithm (RRID:SCR_006763) [24]. Relative expression data were then exported into Microsoft Excel (RRID:SCR_016137) for computing qMIDS index values based on our previously published qMIDS algorithm [15,17]. No-template controls (NTCs) were prepared by omitting brush sample during RNA purification, and eluates were used as NTCs.

### 2.3. Statistical Analysis

Patient demographics and clinicopathological non-parametric characteristics (frequency) were investigated using Chi-square statistics (https://www.socscistatistics.com/tests/, accessed on 5 March 2026). Statistical *t*-test (*p*-value/−Log*p*; Microsoft Excel) and Mann–Whitney U non-parametric (Z-score; https://www.socscistatistics.com/tests/mannwhitney/, accessed on 5 March 2026) test were used for differential analysis between two groups of data. Pearson correlation (R and corresponding *p*-value/−Log*p*; https://www.socscistatistics.com/tests/pearson/, accessed on 5 March 2026) was used to assess the degree of correlation between two datasets. Receiver operating characteristic (ROC) curves were generated to obtain area under the ROC curves (AUC) to assess diagnostic efficiency (performed using Pivot-table method within Microsoft Excel, and DeLong test using the pROC package [25] in R version 4.5.2; The R Foundation for Statistical Computing). Principal Component Analysis (PCA, https://www.statskingdom.com/pca-calculator.html, accessed on 5 March 2026) was used to visualize the biomarker clustering patterns amongst different sample cohorts. Kruskal–Wallis ANOVA with Bonferroni correction (https://www.statskingdom.com/kruskal-wallis-calculator.html, accessed on 5 March 2026) was used to test multiple groups (qMIDS^NPC^ Index on different NPC staging) of data. Diagnostic test performance based on cut-off values was calculated using a Diagnostic Test Calculator [26]. Scattered box–whisker dot plots were created in R using the Beeswarm package (https://github.com/aroneklund/beeswarm, accessed on 5 March 2026) [27].

### 2.4. Data Availability

The data generated in this study are not available publicly to protect patient privacy; however, deidentified data are available upon reasonable request from the corresponding author.

## 3. Results

### 3.1. Participants Demographics and NPC Clinicopathological Characteristics

In the current cohort, no statistical difference in age (Figure 1A) was found between NPM and NPC patients. The overall mean age of NPC patients was 45.1 ± 13.1 (mean ± SD), compared to 47.1 ± 17.9 in participants donating NPM. Also, there was no difference in age distribution found between females and males with NPM or NPC. However, within females with NPC, we found that the patients with recurrent/metastatic tumors (29.3 ± 6.7) were 1.6-times younger than those with non-recurrent tumors (47.5 ± 8.1; *p* = 0.03; see Figure 1A,C). No significant difference was found between males with and without recurrent/metastatic tumors. We next investigated the incidence of NPC between females and males and found 4.9-fold more NPC cases in males (n = 34) than females (n = 7), with a Chi-square statistic of a marginal significance (*p* = 0.07; Figure 1B). Within all NPC patients, we further investigated the incidence of non-recurrent vs. recurrent/metastatic tumor occurrence in different tumor stages and found that there was a significant difference (Chi-square, *p* = 0.002) in tumor-stage distribution between non-recurring and recurring/metastatic NPC. As expected, non-recurring tumors were predominantly with stage I–III, whilst recurring/metastatic tumors were predominantly with stage IVa and IVb (Figure 1B,D). However, we did not find significant difference between non-recurring and recurring/metastatic patients with or without family history of NPC (Figure 1B,E). We also did not find significant difference in EBV DNA levels (copies/mL) between non-recurring and recurring/metastatic patients (Figure 1B,F).

### 3.2. Molecular Profiles of OSCC Biomarkers in NPC

**Figure 2:**
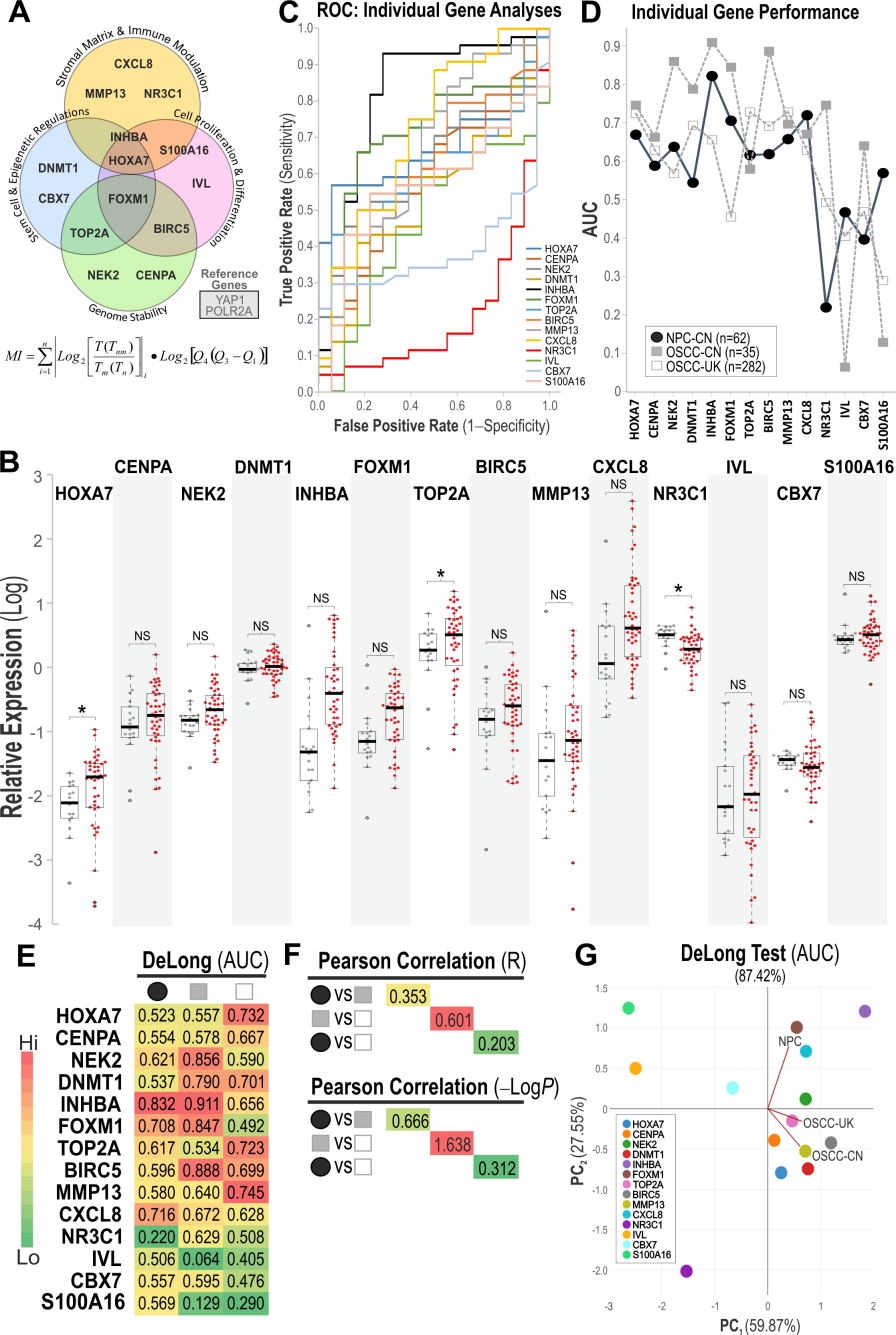
Differential mRNA expression characteristics of OSCC biomarkers in NPM and NPC. (**A**) qMIDS^V2^ biomarker panel consisting of 14 target genes with functional significance in the regulation of the stroma and tumor microenvironment, stem cell and epigenetics, genome stability, and cell proliferation and differentiation [15,17]. Two reference genes were used to calculate the relative target gene expression, and the qMIDS algorithm was used to compute the qMIDS index for cancer diagnosis [15]. (**B**) Relative gene expression profiles between NPM (grey symbols) and NPC (red symbols) for each target gene. Student’s *t*-test * *p* < 0.05 indicates statistically significant difference; NS, not significant. (**C**) Individual gene ROC analyses to investigate the diagnostic performance of each gene in differentiating between NPM and NPC. (**D**) Individual gene performance (AUC) in NPC (solid black circle symbols) and two geographically independent cohorts of OSCC from China (CN—grey square symbols) and United Kingdom (UK—open square symbols) obtained from previous study [17]. (**E**) Heatmap AUC from DeLong test (see Supplementary File for individual marker test results) of individual gene in NPC (black solid circle), OSCC-CN (grey square), and OSCC-UK (white square), with respective Pearson correlation (R) values between each cohort tabulated in (**F**), Biomarkers PCA plots for the 3 cohorts based on (**G**), and DeLong Test AUC values with cohort clustering direction shown in annotated red lines.

Relative quantification of 14 target genes (*HOXA7, CENPA, NEK2, DNMT1, INHBA, FOXM1, TOP2A, BIRC5, MMP13, CXCL8, IVL, NR3C1, CBX7,* and *S100A16*), with functional significance in the regulation of the stroma and tumor microenvironment, stem-cell and epigenetics, genome stability, and cell proliferation and differentiation [15,17], against two reference genes (*YAP1* and *POLR2A*; Figure 2A), was performed on all NPM and NPC samples in this study. Individual relative gene expression profiles are shown in Figure 2B. Of the 14 target genes, only three genes (*HOXA7, TOP2A*, and *NR3C1*) were found to be differentially expressed between NPM and NPC (*p* < 0.05). We subsequently investigated using ROC/AUC analyses to investigate individual gene performance in segregating NPM and NPC (Figure 2C), with their corresponding AUC values plotted in Figure 2D and tabulated in Figure 2E. Additionally, we also used the DeLong test because it is a non-parametric method specifically designed to assess the statistical significance of ROC-derived AUC values without relying on distributional assumptions. Applying the DeLong test yielded AUC significance results (see Supplementary File for detail marker test results) that closely matched our original analysis, confirming that the diagnostic patterns and overall conclusions remained consistent.

**Figure 3:**
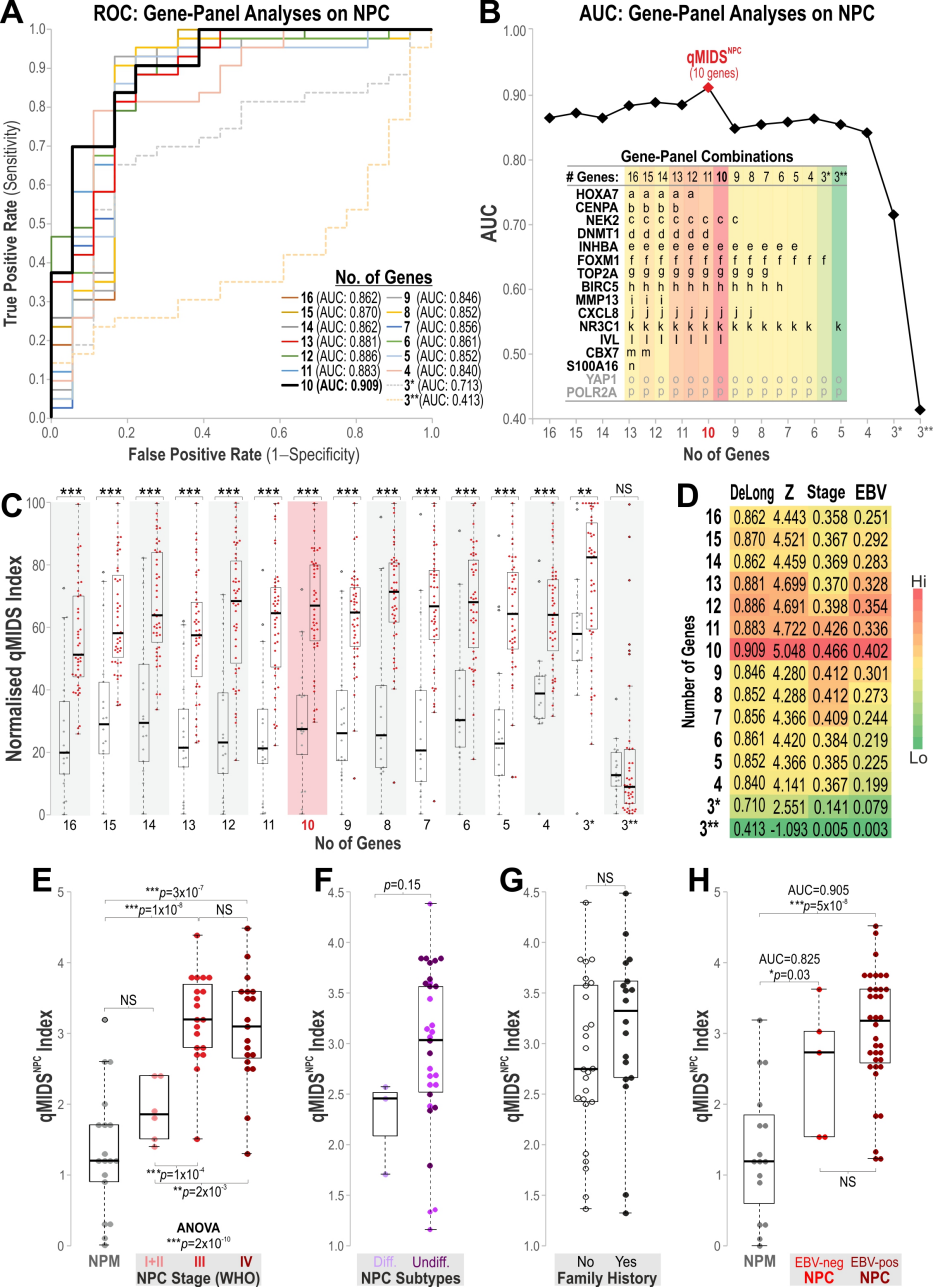
qMIDS test performance characterization and optimization for NPC. (**A**) Gene-panel ROC analyses on NPC cohort to investigate the qMIDS test performances across gene combinations from 16 down to 3 genes with corresponding AUC values listed within the figure. (**B**) AUC data from panel (**A**) are plotted against the number of genes, with an inset table showing corresponding gene combinations with heatmap color shades applied (see (**D**)). The 16-gene panel consists of 14 target genes and 2 internal reference genes (*YAP1* and *POLR2A*). The 10-gene combination was found to be optimum—this 10-gene combination was subsequently named qMIDS^NPC^. (**C**) Individual qMIDS data distribution of each gene panel combination from 16 to 3 genes in NPM (grey symbols) and NPC (red symbols). The 10-gene (qMIDS^NPC^) panel is indicated in red shaded box. Datapoints representing corresponding gene-panel qMIDS index values plotted as Beeswarm scattered box–whisker dot plots (box horizontal lines represent median and 25–75% percentiles, whiskers represent lowest and highest values, and outliers are beyond the whiskers). Student’s *t*-test * *p* < 0.05, ** *p* < 0.01 and *** *p* < 0.001 indicates statistically significant difference; NS, not significant. (**D**) Heatmap (colour shades applied) summarizing DeLong/AUC (see Supplementary File for detail gene-panel titration test results), Mann–Whitney U (Z) values, and linear regression correlation with EBV levels (r^2^ values; see also panel (**H**) below) of corresponding gene panel combinations. (**E**) qMIDS^NPC^ Beeswarm scattered box–whisker dot plots showing data distribution for NPM and NPC stages (I + II, III and IV). (**F**) qMIDS^NPC^ data distribution categorized as differentiated and undifferentiated (poorly differentiated, light purple; and undifferentiated, dark purple symbols). (**G**) qMIDS^NPC^ data distribution of NPC without (open symbols) and with (close symbols) previous family history of NPC. NS, not significant. (**H**) qMIDS^NPC^ data distribution for NPM and EBV-negative and EBV-positive NPC. Statistical *t*-test *p*-values and corresponding AUC values are indicated within the figure.

*INHBA* was found to perform best (AUC = 0.822), followed by *CXCL8* (AUC = 0.720) and *FOXM1* (AUC = 0.706). The differential behavior of these three genes highlights their probable contribution as driver markers of NPC. This is reinforced by the fact that they were later found to be retained in the optimized gene combination that delivered the strongest overall diagnostic accuracy (Figure 3). Next, we questioned if these gene performances in NPC were similar to OSCC given that both originated from mucosal epithelium. We therefore analyzed and compared the same set of biomarkers measured in two geographically independent OSCC cohorts from China (OSCC-CN) and the United Kingdom (OSCC-UK) published previously [17]. In the OSCC-CN cohort, *INHBA* was found to perform best (AUC = 0.909), followed by *BIRC5* (AUC = 0.886), *NEK2* (AUC = 0.860), and *FOXM1* (AUC = 0.845). Interestingly, in the OSCC-UK cohort, *TOP2A* and *MMP13* (both AUC = 0.729) were best performing, followed by *HOXA7* (AUC = 0.724). Note that the lower AUC levels of the genes in the OSCC-UK cohort could be due to the larger sample size (n = 282), as compared to NPC (n = 62) and OSCC-CN (n = 35).

We further assessed inter-cohort similarity by performing Pearson correlation analyses on gene-panel performance metrics, specifically comparing gene-specific DeLong AUC values and *t*-test (−Log*p*) statistics across cohorts (Figure 2F). As expected, a statistically significant correlation was observed only between the OSCC-CN and OSCC-UK cohorts, indicating that these two oral cancer cohorts share closely aligned biomarker signatures. This finding was reinforced by PCA analyses based on both DeLong AUC and *t*-test (−Log*p*) values, which demonstrated that the NPC cohort exhibits a distinct gene-performance profile clearly separable from both OSCC cohorts, whereas the two OSCC cohorts—despite originating from different geographical regions (China and the United Kingdom)—clustered tightly together (Figure 2G). Results derived from ROC analysis were highly comparable with those derived from the DeLong test (see Supplementary File). This clear separation of NPC from OSCC cohorts therefore provided the rationale to systematically assess which combinations of biomarkers would yield the most robust diagnostic performance for NPC.

### 3.3. qMIDS Diagnostic Test Optimization for NPC Detection

We previously validated our qMIDS algorithm in an international multicohort study that included 535 OSCC samples, which compute from a panel of 16 biomarker gene expression levels into a clinically useful ‘Malignancy Index’ for OSCC diagnosis and prognosis, demonstrating that our qMIDS algorithm could unveil otherwise hidden biomarker signatures based on a panel of carefully curated genes [17]. In this study, we hypothesized that the same 16-biomarker panel or a subset of this could be used as a diagnostic and/or prognostic tool for NPC risk stratification. Given that the NPC molecular profile was found to be different but shared with those in OSCC (Figure 2), and in order to identify the best-performing gene-panel combination for NPC, we performed gene panel-titration analyses by removing one gene at a time and measured test performance using ROC/AUC analyses on each gene panel, from 16-gene down to three-gene combinations (Figure 3A). To quantify the contribution of each gene to overall classifier performance, we systematically evaluated all possible single-gene omissions using a stepwise titration strategy. Starting with the full 16-gene panel (AUC = 0.862; Figure 3A,B), we removed each gene individually and recalculated the corresponding AUC values. The gene whose removal resulted in the smallest decrease in AUC was considered the least contributory and therefore designated as dispensable. Using this criterion, *S100A16* emerged as the first gene to be removed, followed sequentially by *CBX7*, *MMP13*, *CENPA*, *HOX7A*, *DNMT1, IVL, NEK2, CXCL8, TOP2A, BIRC3, INHBA, FOXM1,* and *NR3C1*. The complete elimination sequence and its effect on AUC are presented in Figure 3B, where the inset table illustrates all gene-panel combinations assessed during this iterative reduction process. Comparing the two final three-gene combinations revealed that the FOXM1-based panel retained substantially stronger diagnostic performance, with FOXM1 alone achieving an AUC of 0.713, whereas *NR3C1* alone produced a markedly lower AUC of 0.413. Overall, the 10-gene panel (containing eight target genes, i.e., *NEK2, INHBA, FOXM1, TOP2A, BIRC5, CXCL8, NR3C1*, and *IVL*, relative to two reference genes, *YAP1* and *POLR2A*) showed the best biomarker panel performance in segregating NPM from NPC, with an AUC of 0.909 (Figure 3B,C).

To assess the statistical significance of differences in gene-panel performance, we evaluated AUC values using ROC analysis and the DeLong test (see also Supplementary File), together with the Mann–Whitney U (Z) test. Because AUC is fundamentally based on the ranked ordering of classifier scores, both the DeLong and Mann–Whitney U tests provide non-parametric measures of discrimination that do not rely on distributional assumptions. The close agreement between parametric and non-parametric test results indicates that the sampling distribution of AUC values was approximately normal for this dataset (Figure 3C,D and Supplementary File). Using both testing frameworks strengthened the robustness of our conclusions by ensuring that significance estimates were consistent across complementary parametric and non-parametric approaches. These findings were in strong concordance with the AUC analysis, confirming that the 10-gene panel exhibited the best overall performance (Figure 3D), and we subsequently designated this optimized panel as qMIDS^NPC^. Among all gene-panel combinations, EBV DNA levels showed the strongest—though still modest—correlation with qMIDS^NPC^ (R^2^ = 0.405; Figure 3D).

### 3.4. Prognostic Potential of qMIDS^NPC^ for Detecting NPC Independent of EBV Status

We subsequently investigated the prognostic potential of qMIDS^NPC^ for differentiating between different NPC tumor stages. Whilst qMIDS^NPC^ index values were not significantly different between NPM and NPC stage I + II, it could significantly differentiate between NPM and stage III (*p* = 2 × 10^−8^) and IV (*p* = 3 × 10^−7^). Furthermore, qMIDS^NPC^ also significantly distinguished stage I + II from stage III (*p* = 1 × 10^−4^) and IV (*p* = 2 × 10^−3^). Multi-group comparison using Kruskal–Wallis ANOVA amongst all tumor stages of NPC showed an overall significant difference between staging (*p* = 2 × 10^−10^; Figure 3E), confirming the prognostic potential of qMIDS^NPC^ for NPC risk stratification. We further investigated if qMIDS^NPC^ could differentiate between different histopathological subtypes of NPC, and we found a difference between differentiated and undifferentiated NPC. Although undifferentiated NPC had a higher qMIDS index, this was not statistically significant (*p* = 0.15; Figure 3F), probably due to the limited sample size. We also found no significant difference between samples with and without family history of NPC (Figure 3G). Interestingly, qMIDS^NPC^ could significantly differentiate NPM from both EBV-negative NPC (*p* = 0.03, AUC = 0.825) and EBV-positive NPC (*p* = 5 × 10^−8^, AUC = 0.905; Figure 3H).

### 3.5. qMIDS^NPC^ Diagnostic and Prognostic Test Performances on NPC

**Figure 4:**
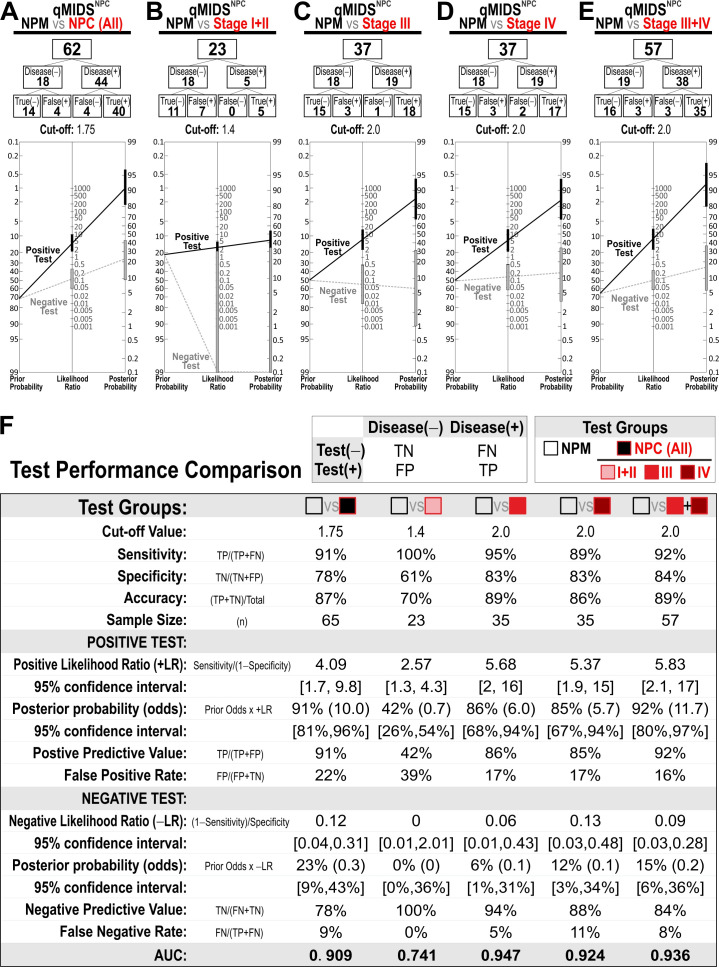
qMIDS^NPC^ prognostic test performance analyses for different tumor stages of NPC. qMIDS^NPC^ comparisons for (**A**) NPM vs. all NPC; (**B**) NPM vs. NPC stage I + II; (**C**) NPM vs. NPC stage III; (**D**) NPM vs. NPC stage IV; and (**E**) NPM vs. NPC stage III + IV. (**F**) Test performance analyses comparison table showing respective test results.

Given both the diagnostic and prognostic potentials of qMIDS^NPC^ observed above (Figure 2 and Figure 3), we subsequently investigated its test performances in segregating NPM from NPC and different NPC tumor stages. An optimum cut-off value that produced the lowest false-positive and -negative rates between each pair of data comparisons was applied (Figure 4A–E), and respective test performance was calculated; and their results are summarized in Figure 4F. Using qMIDS^NPC^ as a diagnostic test for differentiating between NPM and NPC (Figure 4A), at an optimum cut-off at 1.75, the test performance was 91% sensitivity, 78% specificity, 87% accuracy, 91% positive predictive value (PPV), and 78% negative predictive value (NPV; Figure 4F). Due to the small sample size of stage I + II (n = 6), the diagnostic performance of qMIDS^NPC^ in this subgroup may be less reliable and should therefore be interpreted with caution.

Using qMIDS^NPC^ as a prognostic test for NPC risk stratification between NPM and NPC stage I + II (Figure 4B), at an optimum cut-off at 1.4, the test performance was 100% sensitivity, 61% specificity, 70% accuracy, 42% PPV, and 100% NPV (Figure 4F). Between NPM and NPC stage III (Figure 4C), at an optimum cut-off at 2.0, the test performance was 95% sensitivity, 83% specificity, 89% accuracy, 86% PPV, and 94% NPV (Figure 4F). Between NPM and NPC stage IV (Figure 4D), at an optimum cut-off at 2.0, the test performance was 89% sensitivity, 83% specificity, 86% accuracy, 85% PPV, and 88% NPV (Figure 4F). Between NPM and NPC stage III + IV (Figure 4E), at an optimum cut-off at 2.0, the test performance was 92% sensitivity, 84% specificity, 89% accuracy, 92% PPV, and 84% NPV (Figure 4F). Overall, the best prognostic test performance was found to be between NPM and stage III, a finding which is in agreement with its AUC of 0.947 being the best amongst the tested groups (Figure 4F).

## 4. Discussion

The qMIDS^V2^ test, consisting of 16 mRNA biomarkers (14 targets and two reference genes), was originally developed for risk stratification in HNSCC [15] and later validated in three geographically distinct OSCC cohorts from UK, China and India, confirming the diagnostic and prognostic values of the 16 mRNA biomarkers for squamous cancer detection and risk stratification [17]. Unlike HNSCC, NPC is known for its higher potency to metastasize to distant sites and its unique response to treatment. While plasma EBV DNA is widely recognized as a reliable marker for NPC screening, there is still no effective biomarker for early detection and risk stratification in EBV-negative NPC patients. Aiming to explore an EBV-independent maker, we repurpose qMIDS^V2^ test for detecting NPC in the current study, we measured the same 16 biomarkers in a Chinese cohort donating 18 NPM and 44 NPC tissue samples. Initially, individual gene expression profiles revealed only three genes (HOXA7, TOP2A, and NR3C1) were found to be differentially expressed between NPM and NPC. Further ROC/AUC analyses on individual target genes reveal that the current Chinese NPC cohort exhibited a shared but distinct molecular signature to two of our previously studied OSCC cohorts from China and the United Kingdom which clustered together. This indicates that although NPC and OSCC are both originated from squamous epithelium, the NPC molecular profile was found to be shared but distinguishable from OSCC. This supports a transcriptome network analysis finding that NPC and OSCC shared functional transcriptomics modules [28]. Despite the molecular differences, our qMIDS algorithm was able to calculate a clinically meaningful ‘Malignancy Index’ from a unique subset of 10 genes (containing eight target genes, namely *NEK2, INHBA, FOXM1, TOP2A, BIRC5, CXCL8, NR3C1,* and *IVL*, relative to two reference genes, *YAP1* and *POLR2A*, collectively named qMIDS^NPC^), that produced the best overall diagnostic performance for the detection of NPC, with AUC = 0.909, 91% PPV, and 78% NPV. Furthermore, we have demonstrated prognostic value for qMIDS^NPC^ in NPC stage stratification, whereby higher index values predicted advanced stages (III and IV) of NPC stages, with AUC = 0.936, 92% PPV, and 84% NPV. Interestingly, EBV DNA levels showed a mediocre correlation with qMIDS^NPC^ (R^2^ = 0.405) but were able to significantly differentiate NPM from both EBV-negative NPC (AUC = 0.825) and EBV-positive NPC (AUC = 0.905), demonstrating that qMIDS^NPC^ retains its biomarker value independent of EBV status. This suggests a partial convergence of molecular mechanism between EBV and a subset of genes within the qMIDS^NPC^. Further studies are required to delineate their shared molecular mechanism in NPC.

The eight target genes have previously been implicated in NPC through diverse oncogenic mechanisms. *NEK2* (never in mitosis gene a-related kinase 2) is linked to centrosome separation and nuclear pore complex dynamics during mitosis, with dysregulation potentially contributing to chromosomal instability, chemoresistance, and poor prognosis in NPC [29,30]. *INHBA* (inhibin subunit beta A) [31], associated with TGF-β signaling, epithelial–mesenchymal transition, and metastasis, has been identified as a risk gene for both NPC and OSCC [28]. *FOXM1* has been linked to chemoresistance [32], the promotion of cell proliferation and stem cell properties in NPC [33], and poor prognosis and metastasis in NPC patients [34,35]. *TOP2A* (DNA topoisomerase II alpha) has been identified in as a hub gene and a poor prognostic factor for NPC [36,37]. *BIRC5* (Baculoviral IAP repeat containing 5 or survivin) has been linked to poor prognosis in NPC [38,39,40,41]. *CXCL8* (C-X-C motif chemokine ligand 8 or IL-8) has been associated with radioresistance and poor prognosis in NPC [42,43,44]. *NR3C1* (nuclear receptor subfamily 3 group C member 1 gene encoding a glucocorticoid receptor) modulates immune responses [45], and IVL (involucrin), a keratinocyte differentiation marker, detects the degree of differentiation in NPC [46,47]. With these target genes, the qMIDS^NPC^ collectively quantifies the degree of deregulations in stem-cell fate, cell cycle, genome stability, differentiation, apoptosis, immune evasion, and tumor microenvironmental interactions in NPC.

An intriguing observation in our cohort was that females with recurrent or metastatic NPC were significantly younger than females with non-recurrent disease. This age disparity may reflect underlying biological or etiological differences between subgroups. Whilst our cohort suggests that younger female patients may harbor a more aggressive molecular subtype of NPC, a genomic profiling study reported sex-specific mutational differences in NPC [48], supporting the possibility of underlying biological mechanisms that vary between males and females. Alternatively, age-related hormonal influences could modulate immune responses or epithelial susceptibility, thereby contributing to differences in disease trajectory. Variation in EBV-related pathogenic pathways may also play a role, as EBV latency patterns, viral load dynamics, and host immune control can differ across demographic groups. Although our current dataset is insufficient to confirm the specific mechanisms underlying this finding, the pronounced age difference suggests that recurrent/metastatic NPC in younger females may arise from a distinct biological or etiological pathway. Future studies incorporating molecular profiling, EBV characterization, and larger multi-center datasets will be essential to clarify these factors.

With a global increase of 45% in the incidence of NPC between 2009 and 2019, and considering the fact that it disproportionately affects high-middle and middle social demographic index (SDI) areas, there is an urgent need for cost-effective, fast, and easily accessible interventions to alleviate NPC burden worldwide [2]. As early patient presentation of NPC could lead to 10-year improved patient survival [49], a minimally invasive test requiring only a tiny amount of cells/tissue such as qMIDS^NPC^ could potentially benefit resource-poor settings by offering ease of sampling (e.g., brush cytology or cytosponge could potentially be deployed to obtain non-invasive cell samples from the nasopharyngeal mucosa) by trained nurses instead of specialists. Also, the ability to preserve mRNA samples at room temperature negates the requirement of expensive cold-chain storage and shipping logistics, thus significantly reducing costs. A key translational consideration is whether the 10-gene qMIDS^NPC^ signature would retain comparable performance when applied to non-invasive swab or brush samples. Although validation on non-invasive cytology samples is ongoing, our prior experience with qMIDS-type assays in brush-based specimens suggests that meaningful diagnostic separation may still be achievable (manuscript in preparation). Furthermore, this non-invasive test allows for routine repeat sampling, enabling long-term disease surveillance with minimal harm to patients. Following the Coronavirus disease 2019 (COVID-19) pandemic, RT-qPCR diagnostic infrastructure has been significantly up-scaled globally to cope with RNA-testing demand [50]. This enables cost-effective, rapid integration and utilization of existing qPCR diagnostics infrastructure. The scalability of fully automatable, high-throughput RT-qPCR processing enables rapid and efficient large-scale sample screening, accelerating decision-making and enhancing overall patient management workflows.

## 5. Limitations

One of the potential limitations in the current study is the small sample size, and the involvement of only a single cohort and single ethnicity (Chinese) limits the scope of this study. As a result, the predictive performance metrics reported here—particularly the PPV and NPV—should be interpreted as estimates that are inherently specific to this single cohort and not currently clinically actionable until further validation is performed on larger cohort. These values are influenced by the underlying disease prevalence and sample composition and therefore cannot be assumed to generalizable to broader clinical populations. While the present findings provide a strong proof of concept for the qMIDS^NPC^ assay, confirmation of PPV and NPV in multi-center studies will be essential to mitigate potential overfitting and to establish the reproducibility and clinical applicability of the optimized gene panel in diverse settings. Future work incorporating larger, geographically distinct cohorts will be critical to validating these diagnostic performance metrics and supporting translation toward clinical use. Nevertheless, all 16 mRNA biomarkers had already been validated previously in ethnically and geographically diverse, and closely related oral mucosa samples from UK, China, and two independent cohorts in India (Uttar Pradesh and Karnataka) [17], and we have demonstrated in this study that a 10 mRNA biomarkers, forming a subset, retain their biomarker power for NPC due to their shared molecular profiles with OSCC. Another limitation of the current study is the lack of a non-malignant disease group (e.g., adenoid hyperplasia [51]) to control for non-specific conditions such as inflammation that could be induced by a myriad of different non-malignant causes (benign growth, trauma, infections, pollutants, autoimmunity, etc.). In our previous OSCC study, all 16 biomarkers were tested on oral lichen planus, an inflammatory lesion with very low malignant potential, and we ruled out inflammation as a confounding factor [17].

Furthermore, the limited ability of qMIDS^NPC^ to significantly distinguish NPM from stage I + II NPC likely reflects both biological and statistical factors. From a biological standpoint, early-stage NPC lesions may retain molecular characteristics that are still closely aligned with normal nasopharyngeal epithelium, resulting in smaller transcriptional shifts and therefore reduced separation in qMIDS^NPC^ scores. This biological proximity is consistent with the notion that early tumor initiation often involves subtle molecular perturbations that may not yet manifest as strongly divergent expression patterns. In addition, the small sample size within the stage I + II subgroup in our cohort inevitably reduces statistical power, increasing variance and limiting the ability to detect modest but potentially meaningful differences. Together, these factors suggest that both early-stage biological similarity and subgroup size constraints contribute to the observed lack of significant discrimination. Due to the small sample size of stage I + II (n = 6), the diagnostic performance of qMIDS^NPC^ in this subgroup may be less reliable and should therefore be interpreted with caution. Larger early-stage cohorts and deeper molecular profiling will be important for determining whether early NPC can be more effectively distinguished from normal tissue in future studies.

## 6. Novelty and Impact Statement

Plasma Epstein–Barr virus (EBV) DNA is a well-established biomarker for nasopharyngeal carcinoma (NPC) detection, risk stratification, and disease surveillance. However, a large number (15–40%) of NPC patients have low-to-undetectable EBV DNA levels, along with non-specific clinical manifestation and anatomical isolation, resulting in late detection and missed treatment opportunities. We have pioneered a novel rapid 1 h mRNA test (qMIDS^NPC^) for NPC risk stratification independent of EBV status, hence providing an alternative or adjunctive test to benefit NPC patients with undetectable or low levels of EBV. Given the high sensitivity of qPCR quantification, with further research and development, the qMIDS^NPC^ test could potentially be rendered non-invasive using swab, brush, or cytosponge sampling methods.

## 7. Conclusions

In this study, we repurposed a subset of 10 genes (qMIDS^NPC^) from a previously validated HNSCC mRNA biomarker panel for both cancer detection and risk stratification in a Chinese NPC cohort by the RT-qPCR method, which generates results within one hour. For cancer detection, qMIDS^NPC^ specifically segregated NPM from NPC, with AUC = 0.909, 91% PPV, and 78% NPV. Meanwhile, for risk stratification, qMIDS^NPC^ predicted advanced NPC stages (III and IV), with AUC = 0.936, 92% PPV, and 84% NPV. qMIDS^NPC^ could also segregate NPM from EBV-negative NPC, with AUC = 0.825. Further larger multicohort studies are required to validate whether the qMIDS^NPC^ test could be used for risk stratification and disease surveillance in patients with undetectable, low, or ambiguous plasma levels of EBV DNA.

## Data Availability

The original contributions presented in this study are included in the article/Supplementary Material. The data generated in this study are not available publicly to protect patient privacy; however, deidentified data are available upon reasonable request from the corresponding author.

## Supplementary Materials

The following supporting information can be downloaded at https://www.mdpi.com/article/10.3390/cancers18081197/s1.

## Abbreviations

AUC, area under the ROC curve; mRNA, messenger RNA; NPM, normal nasopharyngeal mucosa; NPC, nasopharyngeal carcinoma; EBV, Epstein–Barr virus; MIQE, Minimum Information for Publication of Quantitative Real-Time PCR Experiments; NTC, no template control; qMIDS, quantitative Malignancy Index Diagnostic System; QMUL, Queen Mary University of London; RT-qPCR, reverse-transcription quantitative polymerase chain reaction; ROC, receiver operating characteristic; RT, reverse transcription; SCC, squamous cell carcinoma.
